# Apolipoprotein C1 stimulates the malignant process of renal cell carcinoma via the Wnt3a signaling

**DOI:** 10.1186/s12935-020-01713-x

**Published:** 2021-01-11

**Authors:** Hao Jiang, Jing-Yuan Tang, Dong Xue, Yi-Meng Chen, Ting-Chun Wu, Qian-Feng Zhuang, Xiao-Zhou He

**Affiliations:** 1 Department of Urology, The First Affiliated Hospital of Soochow University, Suzhou, People’s Republic of China; 2 Department of Urology, Jiangsu Province Hospital of Chinese Medicine, Affiliated Hospital of Nanjing University of Chinese Medicine, Nanjing, People’s Republic of China; 3 Department of Urology, The Third Affiliated Hospital of Soochow University, 185 Juqian Street, Changzhou, 213003 Jiangsu People’s Republic of China

**Keywords:** APOC1, RCC, Wnt3a

## Abstract

**Background:**

Renal cell carcinoma (RCC) is a clinically common tumor in the urinary system, showing an upward trend of both incidence and mortality. Apolipoprotein C1 (APOC1) has been identified as a vital regulator in tumor progression. This study aims to uncover the biological function of APOC1 in RCC process and the underlying mechanism.

**Methods:**

Differential levels of APOC1 in RCC samples and normal tissues in a downloaded TCGA profile and clinical samples collected in our center were detected by quantitative reverse transcription PCR (qRT-PCR). The prognostic value of APOC1 in RCC was assessed by depicting Kaplan–Meier survival curves. After intervening APOC1 level by transfection of sh-APOC1 or oe-APOC1, changes in phenotypes of RCC cells were examined through CCK-8, colony formation, Transwell assay and flow cytometry. Subsequently, protein levels of EMT-related genes influenced by APOC1 were determined by Western blot. The involvement of the Wnt3a signaling in APOC1-regulated malignant process of RCC was then examined through a series of rescue experiments. Finally, a RCC xenograft model was generated in nude mice, aiming to further clarify the in vivo function of APOC1 in RCC process.

**Results:**

APOC1 was upregulated in RCC samples. Notably, its level was correlated to overall survival of RCC patients, displaying a certain prognostic value. APOC1 was able to stimulate proliferative, migratory and invasive abilities in RCC cells. The Wnt3a signaling was identified to be involved in APOC1-mediated RCC process. Notably, Wnt3a was able to reverse the regulatory effects of APOC1 on RCC cell phenotypes. In vivo knockdown of APOC1 in xenografted nude mice slowed down the growth of RCC.

**Conclusions:**

APOC1 stimulates the malignant process of RCC via targeting the Wnt3a signaling.

## Background

Renal cell carcinoma (RCC) is a prevalent malignancy with relatively high rates of incidence and mortality. It is estimated that there are 65,340 newly onsets of RCC in the United States, including 14,970 deaths [1]. According to the histological classification, clear cell renal cell carcinoma (ccRcc) cases cover approximately 80% of RCC [2, 3]. Metastasis is the major reason for the poor prognosis of RCC. Generally speaking, RCC cases have a poor response to chemotherapy and/or radiotherapy. Radical resection is the best option for RCC patients [4–6]. However, RCC metastases in the early phase remarkably limit the indications for operation, thus resulting in unsatisfactory clinical outcomes [7]. So far, molecular mechanisms underlying RCC metastasis remain largely unclear. Great efforts are required on searching effective and sensitive biomarkers for predicting the metastasis RCC in the early phase, therefore enhancing the therapeutic efficacy on RCC patients.

The human Apolipoproteins are the protein components of chylomicrons, very low-density lipoprotein (VLDL), and high-density lipoprotein (HDL) [8]. Apolipoprotein C1 (APOC1), a member of Apolipoproteins, is located on chromosome 19q13.32 with 6.6 kD [9]. Growing number of evidences have demonstrated the functions of APOC1 in membrane remodeling, cholesterol catabolism, dendritic reorganization and other biological activities [10, 11]. Very recently, APOC1 has been identified as a tumor-associated gene involved in the process of human cancers [9, 12]. It is recognized that lipid peroxidation is a risk factor for the carcinogenesis of RCC [13]. Jong et al. [14] reported that APOC1 contributes to the inhibition of lipoprotein lipase (LPL)-induced hydrolysis of VLDL through the receptor-associated protein-sensitive signaling. The Wnt signaling includes 19 highly conserved secretory glycoproteins that are involved in various cellular processes, such as embryonic development, stem cell maintenance, and carcinogenesis [15]. Very recently, Zhang et al. reported that Apolipoproteins participated in the regulation of cholesterol homeostasis via the Wnt signaling [16]. To our knowledge, potential influence of APOC1 on RCC has not been comprehensively analyzed. This study aims to elucidate the molecular mechanism of APOC1 on regulating RCC process by generating both in vitro and in vivo models. Our findings may guide clinical prevention and treatment of RCC.

## Materials and methods

### Bioinformatics analysis

Expression data of APOC1 in RCC and paracancerous tissues, as well as corresponding clinicopathologic profiles were downloaded from The Cancer Genome Atlas (TCGA) database (https://cancergenome.nih.gov/). Expression differences between cancer and normal tissues were analyzed by edger function, and prognosis was analyzed by survival function. After data processing, expression levels of APOC1 and survival curves of RCC patients were determined.

### Clinical samples of RCC

A total of 20 pairs of RCC and adjacent non-tumoral tissues were collected from January 2010 to August 2014 in the Department of Urology, the Third Affiliated Hospital of Soochow University. TNM staging of RCC was defined based on the Fuhrman histologic grading system. Recruited subjects were followed up through telephone and outpatient review till December 2018, including physical conditions, cancer recurrence, death, etc. No patient received chemotherapy or radiation before surgery and the collected tissues were preserved in liquid nitrogen. All participants read and signed an informed consent, which conformed to standards for the use of human subjects. This study got approval of the Institutional Research Ethics Committee of the Third Affiliated Hospital of Soochow University.

### Cell culture and transfection

RCC cell lines (7860, 769P, ACHN, CAKI-1, OS-RC-2) and the normal human epithelial cells of renal tubules (HK-2) were purchased from the ATCC. Cells were cultivated in a humidified environment with 5% CO_2_ at 37℃. Except for CAKI-1 cells that were cultivated in McCoy’s 5A, the remaining were in RPMI-1640 (GIBCO, Carlsbad, USA). 10% fetal bovine serum (FBS, GIBCO) and 1% penicillin/streptomycin (Invitrogen) were supplemented in the medium.

For the overexpression of APOC1 and Wnt3a, the full cDNA sequences of APOC1 and Wnt3a were synthesized and inserted into pLVX-puro vector to generate pLVX-APOC1, pLVX-Wnt3a, and empty vector was used as negative control. The pLKO vectors containing APOC1 or Wnt3a shRNA sequences were constructed by GeneChem (Shanghai, China). The lentiviruses containing (phU6-EGFP-shRNA-APOC1, sh-Wnt3a, sh-NC, oe-APOC1, oe-Wnt3a and oe-NC) vectors were packaged and provided by GeneChem (Shanghai, China). Lentiviral infection was performed in CAKI-1 and 769P cell lines. Pools of stable transductants were generated by puromycin (4 μg/ml) selection for 2 weeks.

### QRT-PCR

Cells were lysed using TRIzol reagent (Invitrogen, Carlsbad, CA, USA) for isolating RNAs. Qualified RNAs were reversely transcribed into cDNAs using Primescript RT Reagent Kit (Takara, Otsu, Japan), followed by qRT-PCR using SYBR®Premix Ex Taq™ (Takara, Japan). GAPDH was the internal reference. Each sample was performed in triplicate, and relative level was calculated by 2^−ΔΔCt^, and normalized to that of GAPDH. Oligo primers were constructed using Primer 5.0 as follows. APOC1: 5′-GAAGGAGTTTGGAAACACACTG-3′ (forward) and 5′-CATCTTGGCAGAAAGTTCACTC-3′ (reverse); GAPDH: 5′-GAGAGACCCTCACTGCTG-3′ (forward) and 5′-GATGGTACATGACAAGGTGC-3′ (reverse).

### Western blot

Cells or RCC tissues were lysed in RIPA on ice for 15 min, and the mixture was centrifuged for isolating protein samples. The concentration of protein was determined by BCA method. After adjusting protein samples to the same concentration, they were denaturated, separated by SDS-PAGE, and loaded on PVDF membrane. The membrane was cut into small pieces according to the molecular size and blocked in TBST containing 5% skim milk for 2 h. They were incubated with primary (1:1000) and secondary antibodies (1:3000), followed by band exposure using ECL and grey value analyses using ImageJ software. Antibodies were purchased from Abcam (APOC1, Wnt3a, β-Catenin, TCF7, CCND1, Vimentin) and Cell Signaling Technology (GAPDH, MMP2, MMP9, anti-rabbit and anti-mouse secondary antibodies).

### CCK-8 assay

CAKI-1 and 769P cells were inoculated in a 96-well plate with 3 × 10^3^ cells/well. At day 1, 2, 3 and 4, optical density at 450 nm of each sample was recorded using the CCK-8 kit (Dojindo Laboratories, Kumamoto, Japan) for plotting the viability curves.

### Colony formation assay

CAKI-1 and 769P cells were inoculated in the 6-well plate with 1 × 10^3^ cells/well. Medium was replaced once a week in the first week, and twice in the following week. Two weeks later, visible colonies were washed by PBS, fixed in methanol for 20 min and dyed with 0.1% crystal violet (Sigma-Aldrich) for 20 min. After washing in PBS, stained colonies were captured for counting.

### Flow cytometry

Cells were fixed in 70% cold ethanol for 2 h. Later, they were incubated in 100 μL of RNase at 37 °C for 30 min, and 5 μL of AnnexinV-FITC and 5 μL of PI at room temperature in the dark. Cell cycle progression was determined using flow cytometry (FACScan, BD Biosciences) in triplicate.

### Transwell assay

200 μl of serum-free suspension containing 2 × 10^4^ cells and 500 μl of medium containing 10% FBS was respectively added on the top and bottom of a Transwell insert (pore size = 8 μm; Costar, Corning, NY, USA), and cultured for 48 h. Migratory cells on the bottom were induced with methanol for 15 min, and crystal violet for 20 min. Five random fields per sample were selected for capturing using a microscope and cell counting. Invasive cells were examined using Transwell inserts pre-coated with Matrigel (Invitrogen), following the same procedures as abovementioned.

### Tumorigenicity assay

Tumorigenesis assay in nude mice was approved by the Committee on Animal Ethics and Use of Soochow University. Ten female nude mice with 5 weeks old were administrated with 7 × 10^6^ CAKI-1 cells transfected with sh-NC (n = 5) or sh-APOC1 (n = 5), respectively. Briefly, 7 × 10^6^ transfected CAKI-1 cells were diluted in 150 µl of PBS, which was subcutaneously injected into a single side of the posterior flank of each mouse. Tumor width and length were recorded with an interval of one week. Mice were sacrificed at the 6^th^ week for collecting tumor tissues. Tumor volume (mm^3^) was calculated using the formula: Tumor width^2^ × tumor length/2. Immunohistochemistry was performed to evaluate positive expressions of Ki-67, Vimentin and N-cadherin in xenograft tissues.

### Immunohistochemistry (IHC)

Positive expression of APOC1 in RCC tissues was evaluated using the tissue microarray by IHC. Tissue microarray was incubated with the primary antibody at 4℃ overnight and HRP-conjugated secondary antibody on the other day, followed by diaminobenzidine dyeing. IHC staining was independently assessed by two experienced pathologists. RCC tissues were divided into low- and high-staining groups based on IHC staining results for further analysis.

### Statistical processing

SPSS 22.0 was used for statistical analyses and data were expressed as mean ± standard deviation. Differences between groups were compared by the *t*-test. Kaplan–Meier survival curves were depicted to analyze overall survival in RCC patients. The potential influence of APOC1 on clinical features of RCC was analyzed by Chi-square analysis. Each experiment was repeated in triplicate. A significant difference was considered at the level of *p* < 0.05.

## Results

### Expression characteristic and prognostic value of APOC1 in RCC

**Fig. 1:**
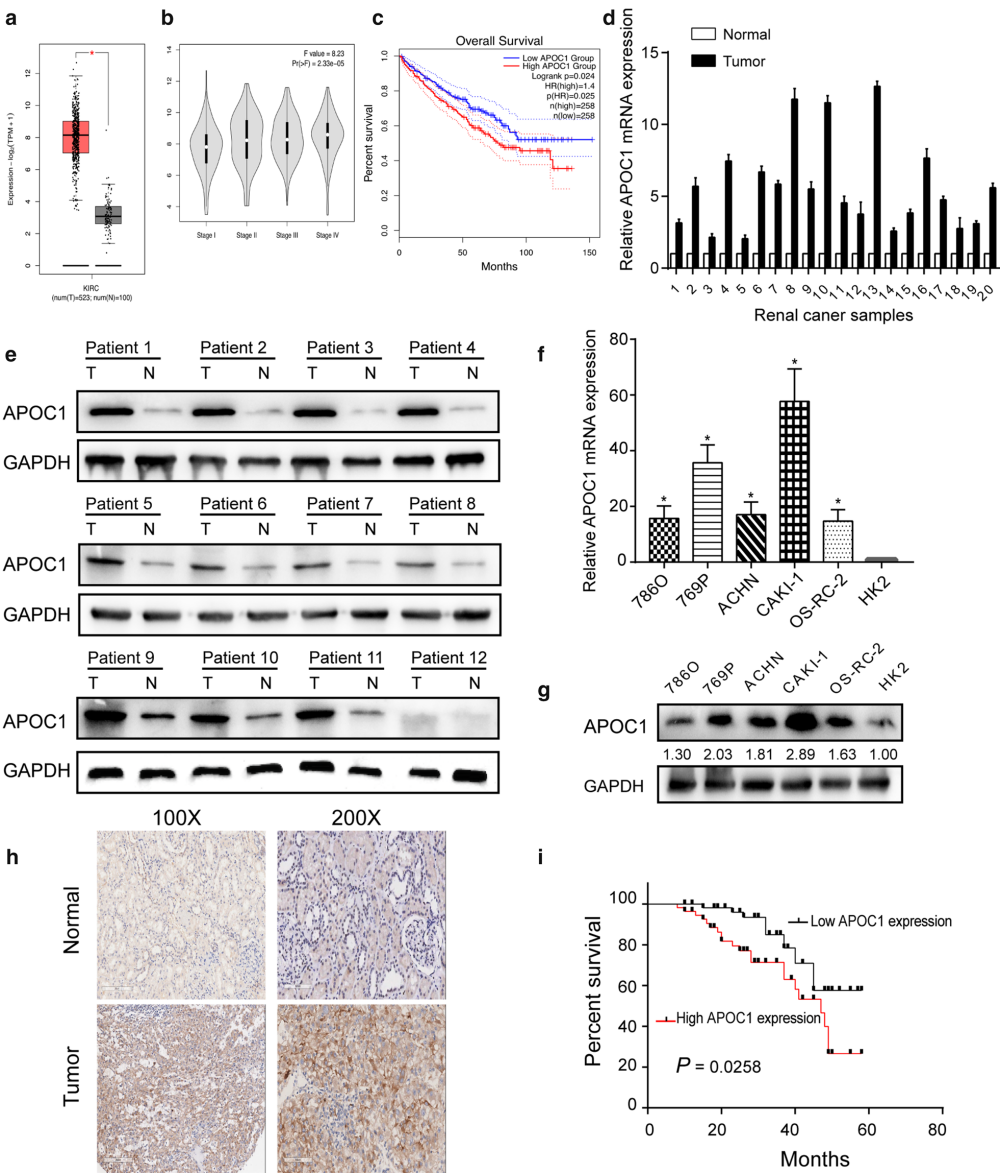
Expression characteristic of APOC1 in RCC. **a** Differential levels of APOC1 in a downloaded TCGA profile containing RCC tissues (n = 523) and normal tissues (n = 100); **b** Differential levels of APOC1 in stage I–IV RCC tissues in TCGA profile; **c** Overall survival in RCC patients based on high or low level of APOC1 in TCGA profile; **d** Differential levels of APOC1 in 20 pairs of RCC and adjacent non-tumoral tissues collected in our center; **e** Protein level of APOC1 in 20 paired cases of RCC and adjacent non-tumoral tissues (12 representative pairs were shown) (T: RCC tissues, N: adjacent non-tumoral tissues); **f** In vitro mRNA level of APOC1 in RCC cell lines; **g** Protein level of APOC1 in RCC cell lines; **h** The relative protein levels of APOC1 were examined by immunohistochemistry in a tissue microarray including 130 pairs of RCC tissues and normal tissues **i** Kaplan–Meier survival curves depicted based on APOC1 level in RCC patients. *P < 0.05, error bars indicate mean ± SD, t test

A profile containing 523 cases of RCC and 100 cases of normal ones was downloaded from TCGA database. It is analyzed that APOC1 was upregulated in RCC tissues, especially advanced tumor cases (Fig. 1a, b). In addition, a worse overall survival was identified in RCC patients expressing high level of APOC1 (Fig. 1c). Subsequently, expression characteristic of APOC1 was examined in clinical samples of RCC collected in our center. As expected, APOC1 was highly expressed in RCC tissues (T) than adjacent non-tumoral ones (N) (Fig. 1d, e and Additional file 1: Figure S1). In vitro level of APOC1 consistently remained higher in RCC cell lines than the control (Fig. 1f, g). To verify the clinical significance of APOC1, IHC in a tissue microarray including 130 cases of RCC was conducted and it revealed the upregulated APOC1 in RCC (Fig. 1h). Kaplan–Meier curves demonstrated that highly expressed APOC1 was unfavorable to the prognosis of RCC (Fig. 1i). Clinical data of RCC patients were analyzed for assessing the clinical significance of APOC1. As Chi-square analysis uncovered, APOC1 level was correlated to histological grade (*P* = 0.027) and TNM staging (*P* = 0.012) in RCC patients, whereas it was unrelated to age, gender, tumor size and histological subtypes (Table 1).

**Table 1:** Basic characteristics of renal cell carcinoma patients and association between the expression of APOC1 and their clinicopathologic characteristics

Variables	Number of cases	APOC1 expression		Chi-square d-test *P*-value
		Low	High	
Age (years)				0.770
≤ 60	80	39	41	
> 60	50	24	26	
Gender				0.592
Male	77	40	37	
Female	53	25	28	
Tumor size (cm)				0.181
≤ 4	61	31	30	
> 4	69	27	42	
Histological subtypes				0.906
Clear cell carcinoma	117	52	65	
Others	13	6	7	
Histological grade				*0.027*
I–II	104	49	55	
III–IV	26	6	20	
TNM staging				*0.012*
I	99	51	48	
II–IV	31	8	23	

### APOC1 stimulated in vitro proliferative ability of RCC

**Fig. 2:**
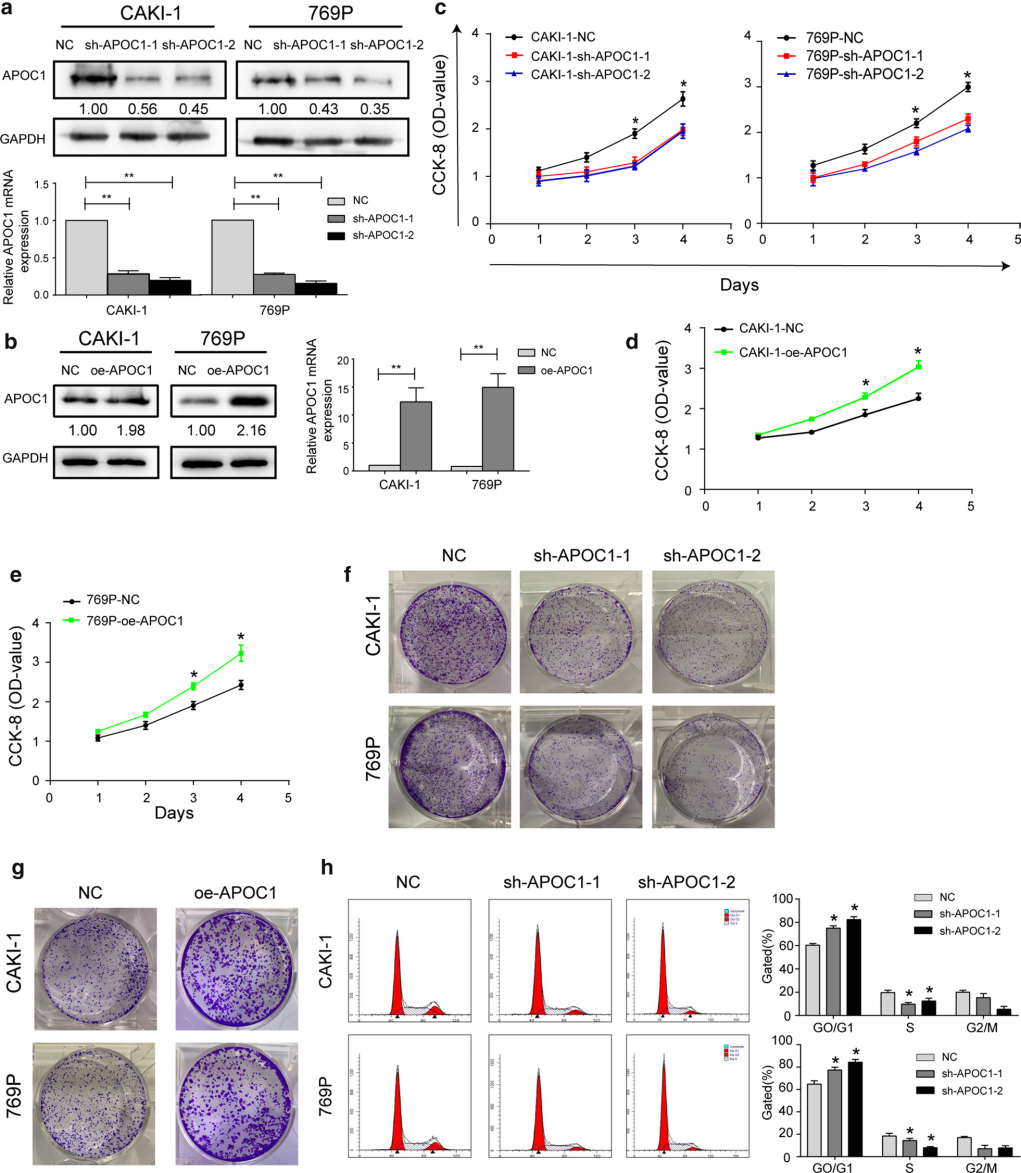
APOC1 stimulated in vitro proliferative ability of RCC. **a** Transfection efficacy of sh-APOC1-1 and sh-APOC1-2 in CAKI-1 and 769P cells. **b** Transfection efficacy of oe-APOC1 in CAKI-1 and 769P cells. **c** Viability in CAKI-1 and 769P cells with APOC1 knockdown. **d**, **e** Viability in CAKI-1 and 769P cells overexpressing APOC1. **f** Colony formation in CAKI-1 and 769P cells with APOC1 knockdown. **g** Colony formation in CAKI-1 and 769P cells overexpressing APOC1. **h** Cell cycle distribution in CAKI-1 and 769P cells with APOC1 knockdown. *P < 0.05, **P < 0.01, error bars indicate mean ± SD, t test

Among the tested RCC cell lines, CAKI-1 and 769P cells with relatively high abundance of APOC1 were utilized in the following experiments. First of all, knock down efficacy of sh-APOC1-1 and sh-APOC1-2 was detected. Either sh-APOC1-1 or sh-APOC1-2 effectively downregulated protein and mRNA levels of APOC1 (Fig. 2a). In addition, transfection efficacy of oe-APOC1 was similarly examined by qRT-PCR and Western blot (Fig. 2b). CCK-8 assay uncovered that knockdown of APOC1 remarkably decreased viability in CAKI-1 and 769P cells, while over-expression of APOC1 promoted the cell proliferation (Fig. 2c–e). Moreover, clonality of RCC cells was attenuated by knockdown of APOC1, and it was stimulated by up-regulation of APOC1 expression (Fig. 2f, g). It is indicated that the proliferative ability of RCC cells was stimulated by APOC1. Flow cytometry was conducted to assess the influence of APOC1 on cell cycle progression of RCC cells. It is shown that cell ratio of G0/G1 phase increased and that of S phase decreased after knockdown of APOC1 in RCC cells (Fig. 2h).

### APOC1 stimulated in vitro migration and invasion of RCC through regulating EMT

**Fig. 3:**
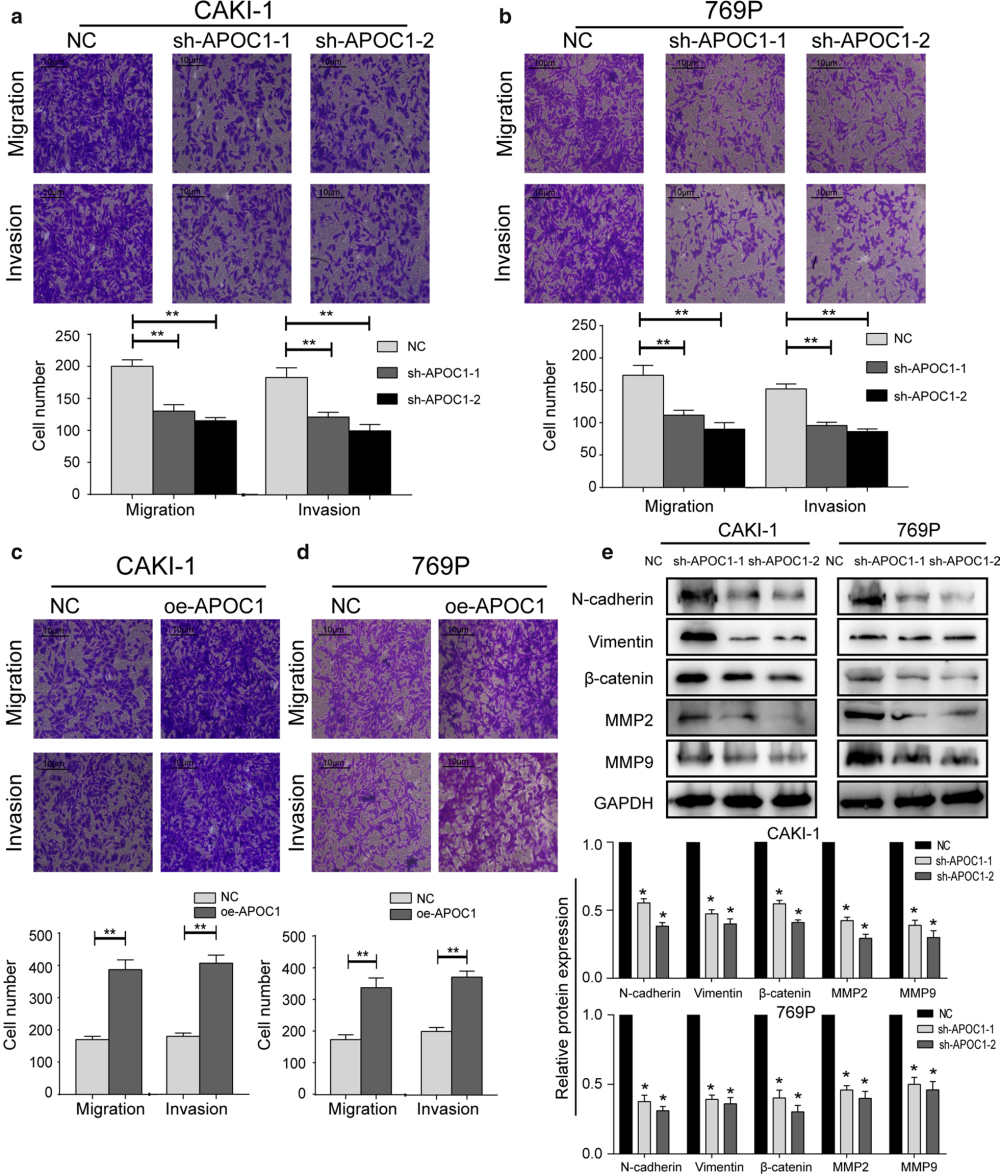
APOC1 stimulated in vitro migration and invasion of RCC through regulating EMT. **a**, **b** Migration and invasion in CAKI-1 and 769P cells with APOC1 knockdown. **c**, **d** Migration and invasion in CAKI-1 and 769P cells overexpressing APOC1. **e** Protein levels of N-cadherin, Vimentin, β-catenin, MMP2, and MMP9 in CAKI-1 and 769P cells with APOC1 knockdown. *P < 0.05, **P < 0.01, error bars indicate mean ± SD, t test

We thereafter explored the migration and invasion of RCC intervened by APOC1 by using transwell assay. Either sh-APOC1-1 or sh-APOC1-2 in CAKI-1 and 769P cells markedly declined numbers of migratory and invasive cells (Fig. 3a, b). Conversely, overexpression of APOC1 strengthened migratory and invasive abilities of RCC cells (Fig. 3c, d). Furthermore, relative levels of EMT proteins were detected by western blot. Knockdown of APOC-1 remarkably downregulated protein levels of N-cadherin, Vimentin, β-catenin, MMP2, and MMP9 in CAKI-1 and 769P cells (Fig. 3e). Taken together, APOC1 was able to stimulate RCC migration and invasion through regulating EMT.

### Wnt3a was partially responsible for APOC1-induced aggravation of RCC

**Fig. 4:**
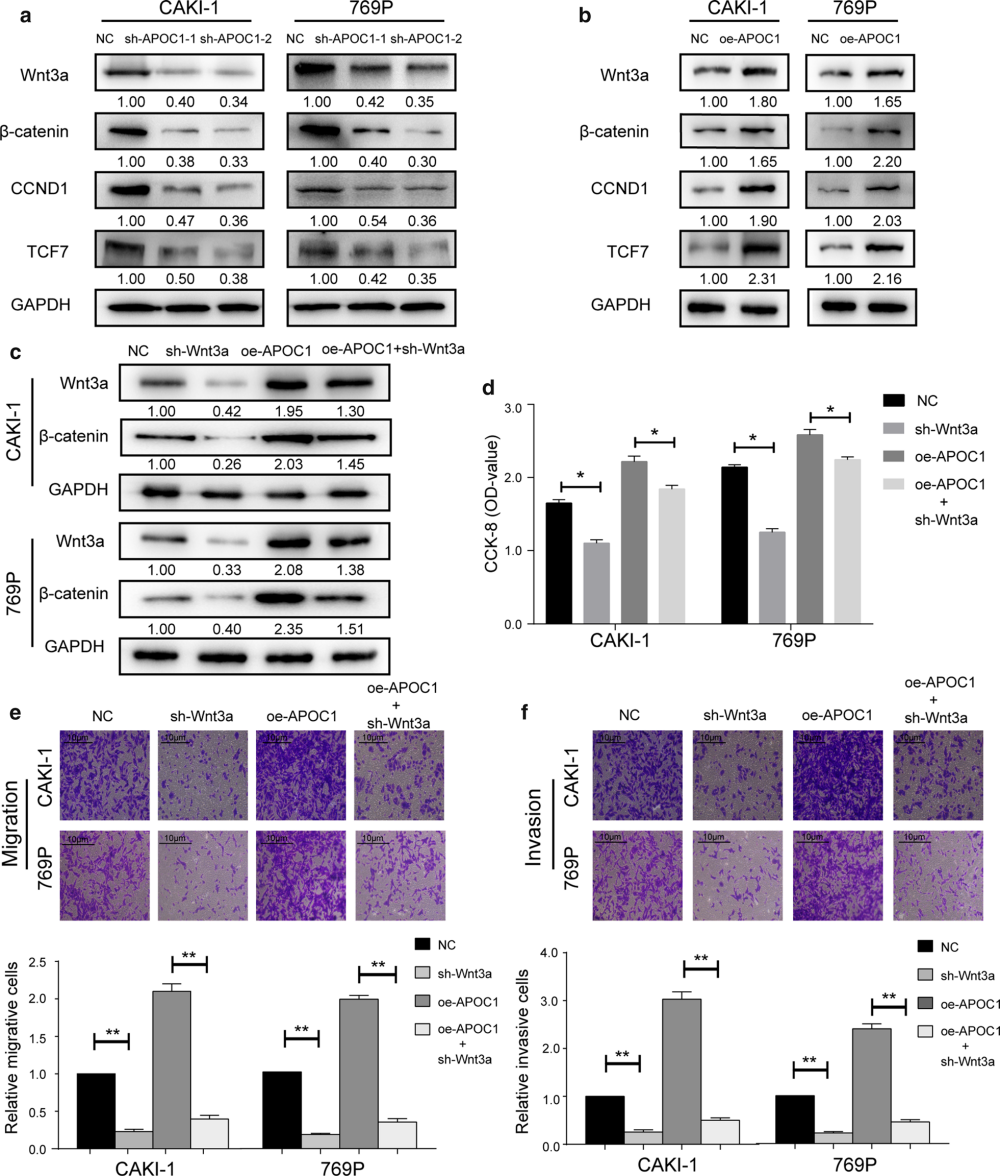
Wnt3a was partially responsible for APOC1-induced aggravation of RCC. **a** Protein levels of Wnt3a, β-catenin, CCND1 and TCF7 in CAKI-1 and 769P cells with APOC1 knockdown. **b** Protein levels of Wnt3a, β-catenin, CCND1 and TCF7 in CAKI-1 and 769P cells overexpressing APOC1. **c** Protein level of Wnt3a and β-catenin in CAKI-1 and 769P cells intervened by Wnt3a and APOC1. **d** Viability in CAKI-1 and 769P cells intervened by Wnt3a and APOC1. **e**, **f** Migration and invasion in CAKI-1 and 769P cells intervened by Wnt3a and APOC1. *P < 0.05, **P < 0.01, error bars indicate mean ± SD, t test

It is well known that the Wnt3a signaling has a close relation to EMT. Here, we detected protein levels of key genes involved in the Wnt3a signaling in CAKI-1 and 769P cells intervened with APOC1. As western blot analyses revealed, knockdown of APOC1 remarkably downregulated protein levels of Wnt3a, β-catenin, CCND1 and TCF7 in RCC cells (Fig. 4a and Additional file 1: Figure S2). On the contrary, their protein levels were upregulated in RCC cells overexpressing APOC1 (Fig. 4b and Additional file 1: Figure S3). Furthermore, we focused on the potential involvement of Wnt3a in APOC1-induced changes of RCC cell phenotypes. Knock down efficacy of sh-Wnt3a was firstly examined. Transfection of sh-Wnt3a not only effectively downregulated Wnt3a and β-catenin in RCC cells, but also reversed the upregulated level of Wnt3a and β-catenin caused by overexpression of APOC1 (Fig. 4c). Furthermore, knockdown of Wnt3a was found to remarkably attenuate proliferative, migratory and invasive abilities of CAKI-1 and 769P cells. Interestingly, the abovementioned phenotypes of RCC cells overexpressing APOC1 were partially reversed by Wnt3a knockdown (Fig. 4d–f). Furthermore, Wnt3a over-expression could reverse the APOC1 knockdown mediated impaired proliferative, migratory and invasive abilities of 769P cell (Additional file 1: Figure S4). To sum up, Wnt3a was at least partially responsible for APOC1-induced aggravation of RCC.

### APOC1 stimulated in vivo growth of RCC

**Fig. 5:**
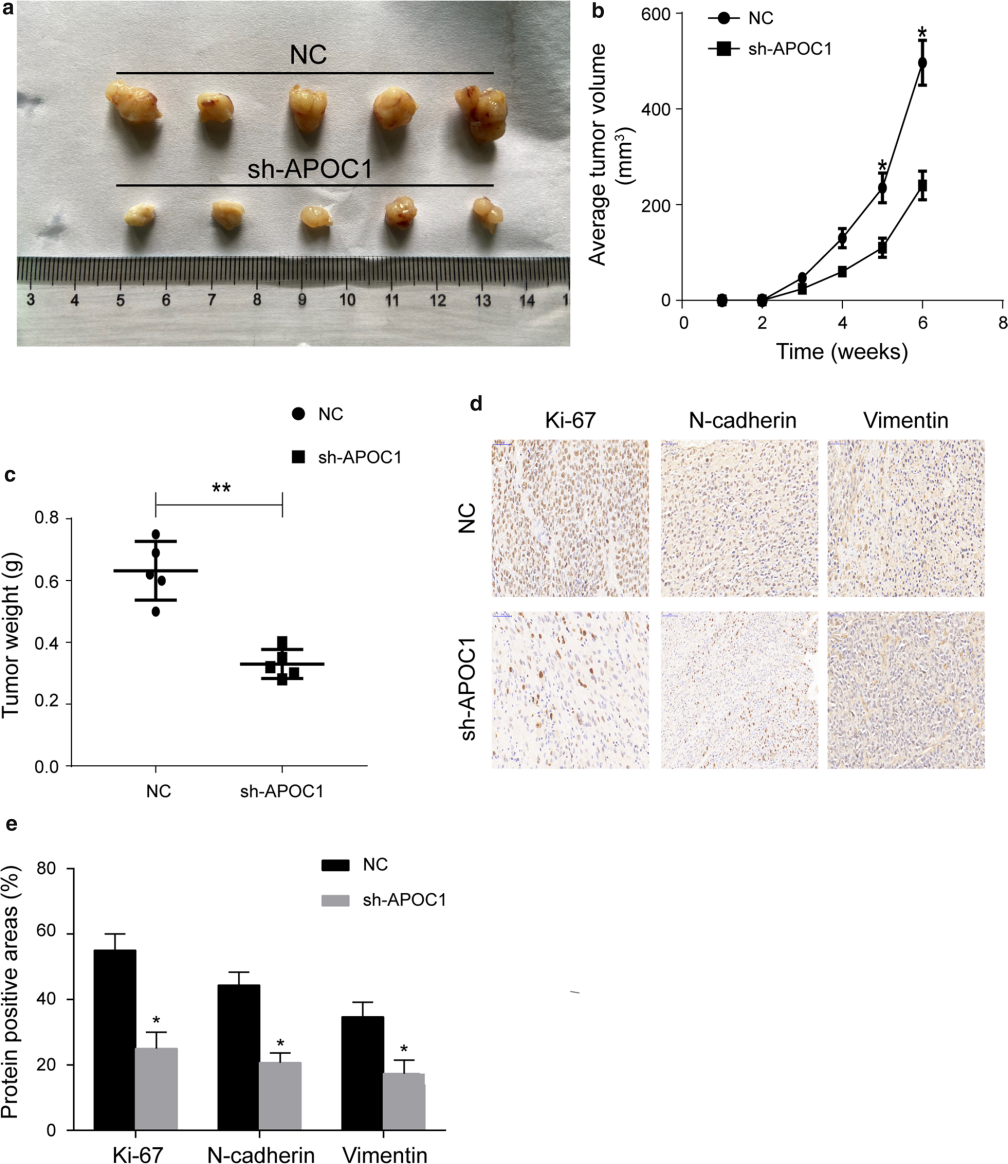
APOC1 stimulated in vivo growth of RCC. Mice were administrated with CAKI-1 cells transfected with sh-NC or sh-APOC1. **a** Representative images of xenografted RCC tissues. **b** Average tumor volume of xenografted RCC tissues. **c** Tumor weight of xenografted RCC tissues. **d** Positive staining of Ki-67, N-cadherin and Vimentin in xenografted RCC tissues. **e** Quantitative analysis for **d**. *P < 0.05, **P < 0.01, error bars indicate mean ± SD, t test

A xenograft model in nude mice was generated to explore the in vivo effect of APOC1 on RCC growth. Mice were administrated with CAKI-1 cells transfected with sh-NC or sh-APOC1 as described in the Method. At the 6^th^ week, mice were sacrificed for harvesting RCC tissues (Fig. 5a). Compared with controls, mice with in vivo knockdown of APOC1 presented a smaller tumor volume and lower tumor weight, suggesting that the growth of RCC was slowed down (Fig. 5b, c). In addition, knockdown of APOC1 suppressed the positive staining of Ki-67 (a proliferation marker), N-cadherin and Vimentin which was consistent with our in vitro findings (Fig. 5d, e).

## Discussion

As a highly prevalent tumor in clinical practice, chemotherapy and radiotherapy for RCC are not as effective as we expect to [17]. At present, individualized therapy based on targeted genes has been well concerned in tumor treatment because of its precise efficacy. Apolipoproteins are responsible for forming lipoproteins and transporting lipids in the circulating system [18]. Functions of lipoproteins vary because of different types of apolipoproteins they contain. In particular, apolipoprotein C exerts an important role in regulating the dynamic metabolism of TRLs. A previous study has demonstrated that a variation of APOC1 increases the susceptibility to RCC, although the specific molecular mechanism is unclear [13]. Our study showed that APOC1 was highly expressed in RCC samples not only in a downloaded RCC profile, but also tumor tissues harvested from RCC patients. In CAKI-1 and 769P cells with APOC1 knockdown, proliferative, migratory and invasive abilities were markedly attenuated. In addition, cell cycle progression of RCC was arrested by APOC1 intervention, which may be attributed to the weakened proliferation. Consistently, in vivo evidences supported our findings that knockdown of APOC1 slowed down the growth rate of RCC in nude mice. We believed that APOC1 was an oncogene involved in RCC process.

Through literature review, APOC1 has been identified as a promising serum marker for human cancers, which is conductive to cancer diagnosis and treatment [19–23]. Su et al. [24] pointed out that APOC1 mediates cell survival, cell cycle distribution and apoptosis of prostate cancer via activating the surviving/Rb/p21/caspase-3 signaling. Ren et al. [9] uncovered that overexpressed APOC1 in colorectal carcinoma predicts a poor prognosis. In pancreatic cancer, serum APOC1 derived from cancer cell serves as a prognostic symbol [25]. Here, Kaplan–Meier survival curves proved the prognostic value of APOC1 in RCC, that is, high level of APOC1 predicted poor overall survival in RCC patients. Li et al. identified that Apoc1 as a novel pro-metastatic factor facilitates the activation of STAT3 and enhances the metastasis of ccRcc cells [26]. This finding is consistent with our results and illustrates an important function of APOC1 in the development and progression of renal cell carcinoma.

EMT is a process in which epithelial cells transform to the mesenchymal phenotype [27]. Expression changes of N-cadherin and vimentin are two typical representatives for EMT [28]. It is well known that EMT is an initial event for acquiring malignant phenotypes. MMPs are functional metalloproteinases for cell behavior mediation. Specifically, MMP2 and MMP9 are considered to be critical in tumor metastasis because of their influences on ECM degradation and tissue remodeling[29]. Western blot analyses in the present study showed that protein levels of EMT-associated genes and MMPs were downregulated in RCC cells with APOC1 knockdown, indicating the capacity of APOC1 in stimulating RCC migration and invasion.

As a classical signaling, the Wnt signaling has been reported to mediate self-renewal and carcinogenesis [30, 31]. Under the normal circumstance, the Wnt signaling helps to maintain the homeostasis through regulating basic cellular functions [32]. A growing number of studies have detected the abnormally activated Wnt signaling during tumor process [33, 34]. The protein encoded by CCND1 belongs to the highly conserved cyclin family, and its members have a significant periodicity of protein abundance throughout the cell cycle progression [35]. CCND1 variation is frequently observed during tumor process [36]. TCF7 encodes a hmg box that contains transcription factors critically involved in the Wnt signaling [37]. Zhan et al. [38] reported that TCF7 participates in the malignant process of nasopharyngeal carcinoma as an oncogene. Consistently, Wu et al. [39] demonstrated that TCF7 accelerates colorectal cancer cells to migrate and invade. In this paper, knockdown of APOC1 remarkably downregulated Wnt3a, β-catenin, CCND1 and TCF7 in RCC cells, whereas overexpression of APOC1 yielded the opposite trends. Furthermore, we found that Wnt3a was able to reverse the regulatory effects of APOC1 on RCC cell phenotypes. It is suggested that APOC1 aggravated the malignant process of RCC at least partially via activation of the Wnt signaling.

## Conclusions

APOC1 is upregulated in RCC samples as an oncogene, which triggers proliferative, migratory and invasive abilities in RCC by activating the Wnt3a signaling. APOC1 is a promising biomarker for RCC, and it can be utilized for developing targeted drugs.

## Supplementary Information

**Additional file 1: Figure S1.** Relative Protein expression of APOC1 in 12 representative paired cases of RCC and adjacent non-tumoral tissues. (T: RCC tissues, N: adjacent non-tumoral tissues), *P < 0.05, error bars indicate mean ± SD, t test. **Figure S2**. Relative Protein expression of Wnt3a, β-catenin, CCND1 and TCF7 in CAKI-1 and 769P cell lines with APOC1 knockdown, *P < 0.05, error bars indicate mean ± SD, t test. **Figure S3.** Relative Protein expression of Wnt3a, β-catenin, CCND1 and TCF7 in CAKI-1 and 769P cell lines overexpressing APOC1, *P < 0.05, error bars indicate mean ± SD, t test. **Figure S4.** Wnt3a was partially responsible for APOC1-induced aggravation of RCC. (a) Protein level of Wnt3a and β-catenin in 769P cell intervened by Wnt3a and APOC1; (b) Viability in 769P cell intervened by Wnt3a and APOC1; (c, d) Migration and invasion in 769P cell intervened by Wnt3a and APOC1. *P < 0.05, **P < 0.01, error bars indicate mean ± SD, t test.

## Data Availability

The authors declare that the data supporting the findings of this study are available within the article.

## Abbreviations

RCC: Renal cell carcinoma
APOC1: Apolipoprotein C1
qRT‐PCR: Quantitative reverse transcription PCR
RIPA: Radio immunoprecipitation assay
BCA: Bicinchoninic acid
SDS-PAGE: Polyacrylamide gel electrophoresis
PVDF: Polyvinylidene fluoride
ECL: Electrochemiluminescence
ATCC: The American Type Culture Collection
KIRC: Kidney renal cell carcinoma
MMP2, MMP9: Matrix metallopeptidase 2 and 9
ccRcc: Clear cell renal cell carcinoma
LPL: Lipoprotein lipase
VLDL: Very low-density lipoprotein
HDL: High-density lipoprotein
EMT: Epithelial-mesenchymal transition
TRLs: Triglyceride-rich lipoproteins
ECM: Extracellular matrix components
IHC: Immunohistochemistry
