# Supplementary figures and images for: Apolipoprotein C1 stimulates the malignant process of renal cell carcinoma via the Wnt3a signaling
Source: Cancer Cell Int. 2021 Jan 11;21:41. doi: 10.1186/s12935-020-01713-x (PMC7802262; doi:10.1186/s12935-020-01713-x)

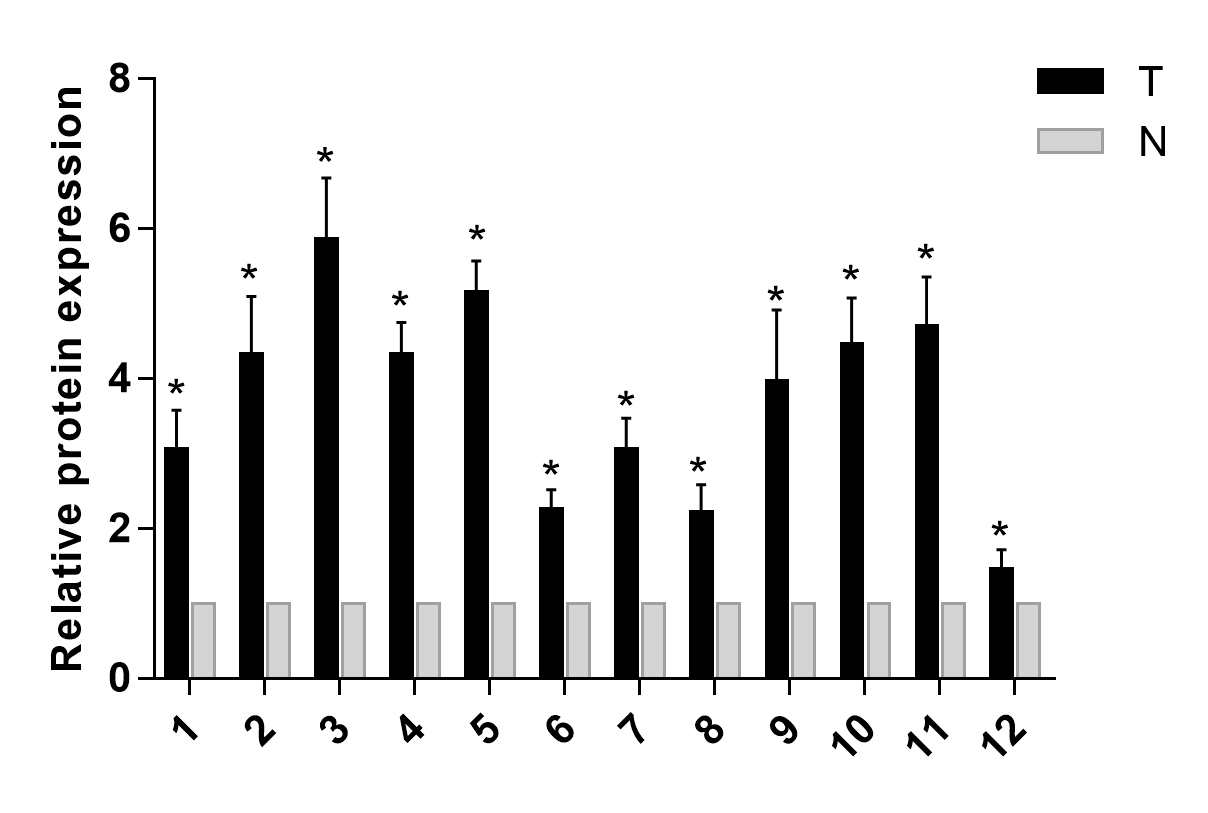

Supplement: Supplementary file 1 — Additional file 1: Figure S1. Relative Protein expression of APOC1 in 12 representative paired cases of RCC and adjacent non-tumoral tissues. (T: RCC tissues, N: adjacent non-tumoral tissues), *P < 0.05, error bars indicate mean ± SD, t test. Figure S2. Relative Protein expression of Wnt3a, β-catenin, CCND1 and TCF7 in CAKI-1 and 769P cell lines with APOC1 knockdown, *P < 0.05, error bars indicate mean ± SD, t test. Figure S3. Relative Protein expression of Wnt3a, β-catenin, CCND1 and TCF7 in CAKI-1 and 769P cell lines overexpressing APOC1, *P < 0.05, error bars indicate mean ± SD, t test. Figure S4. Wnt3a was partially responsible for APOC1-induced aggravation of RCC. (a) Protein level of Wnt3a and β-catenin in 769P cell intervened by Wnt3a and APOC1; (b) Viability in 769P cell intervened by Wnt3a and APOC1; (c, d) Migration and invasion in 769P cell intervened by Wnt3a and APOC1. *P < 0.05, **P < 0.01, error bars indicate mean ± SD, t test. [file 12935_2020_1713_MOESM1_ESM.zip › 12935_2021_1713_MOESM1_ESM.tif]

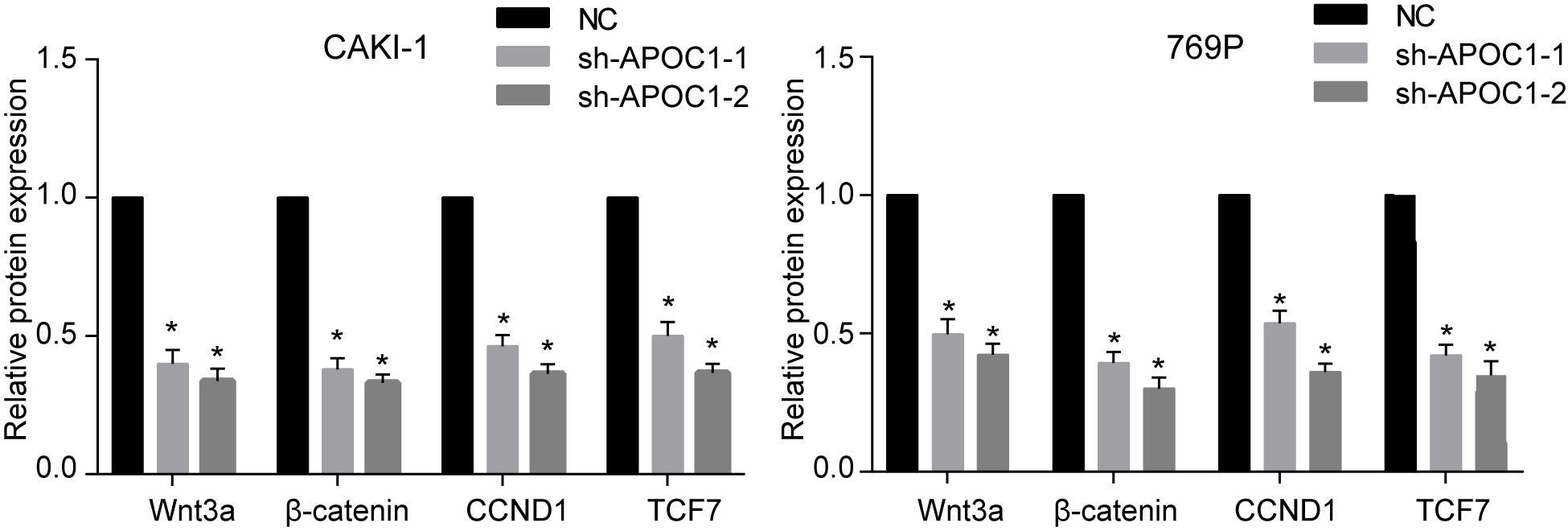

Supplement: Supplementary file 1 — Additional file 1: Figure S1. Relative Protein expression of APOC1 in 12 representative paired cases of RCC and adjacent non-tumoral tissues. (T: RCC tissues, N: adjacent non-tumoral tissues), *P < 0.05, error bars indicate mean ± SD, t test. Figure S2. Relative Protein expression of Wnt3a, β-catenin, CCND1 and TCF7 in CAKI-1 and 769P cell lines with APOC1 knockdown, *P < 0.05, error bars indicate mean ± SD, t test. Figure S3. Relative Protein expression of Wnt3a, β-catenin, CCND1 and TCF7 in CAKI-1 and 769P cell lines overexpressing APOC1, *P < 0.05, error bars indicate mean ± SD, t test. Figure S4. Wnt3a was partially responsible for APOC1-induced aggravation of RCC. (a) Protein level of Wnt3a and β-catenin in 769P cell intervened by Wnt3a and APOC1; (b) Viability in 769P cell intervened by Wnt3a and APOC1; (c, d) Migration and invasion in 769P cell intervened by Wnt3a and APOC1. *P < 0.05, **P < 0.01, error bars indicate mean ± SD, t test. [file 12935_2020_1713_MOESM1_ESM.zip › 12935_2021_1713_MOESM2_ESM.tif]

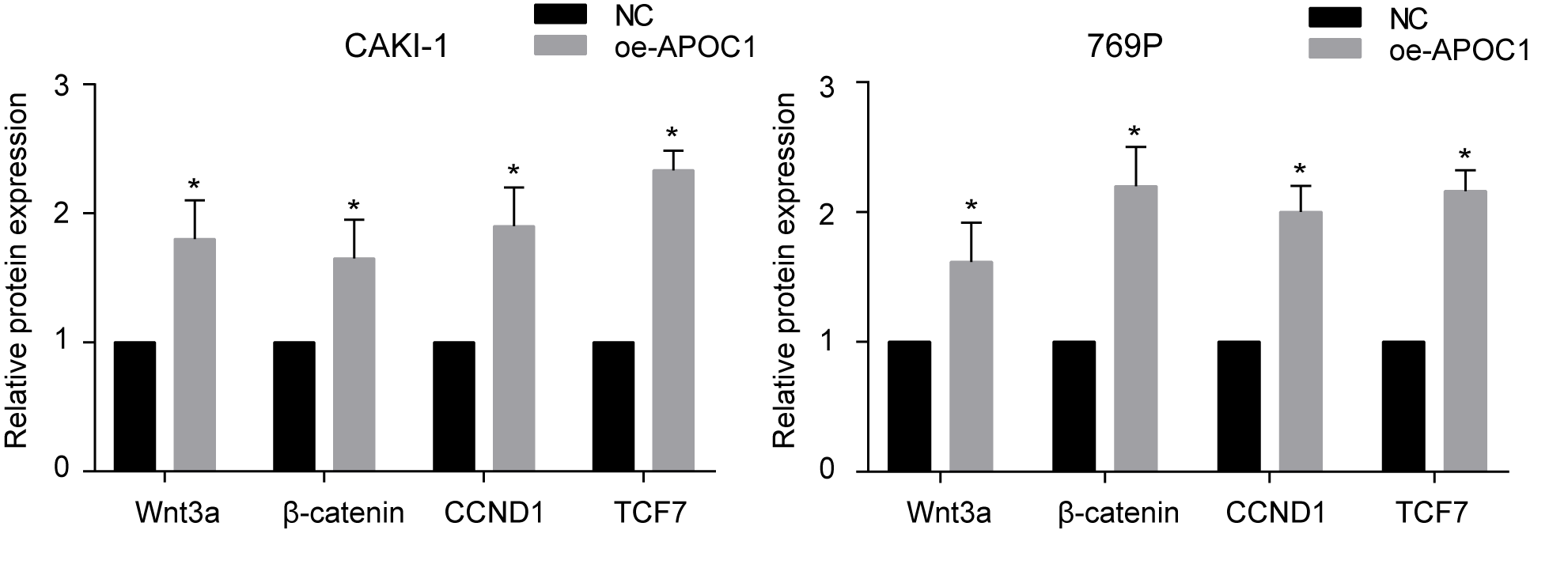

Supplement: Supplementary file 1 — Additional file 1: Figure S1. Relative Protein expression of APOC1 in 12 representative paired cases of RCC and adjacent non-tumoral tissues. (T: RCC tissues, N: adjacent non-tumoral tissues), *P < 0.05, error bars indicate mean ± SD, t test. Figure S2. Relative Protein expression of Wnt3a, β-catenin, CCND1 and TCF7 in CAKI-1 and 769P cell lines with APOC1 knockdown, *P < 0.05, error bars indicate mean ± SD, t test. Figure S3. Relative Protein expression of Wnt3a, β-catenin, CCND1 and TCF7 in CAKI-1 and 769P cell lines overexpressing APOC1, *P < 0.05, error bars indicate mean ± SD, t test. Figure S4. Wnt3a was partially responsible for APOC1-induced aggravation of RCC. (a) Protein level of Wnt3a and β-catenin in 769P cell intervened by Wnt3a and APOC1; (b) Viability in 769P cell intervened by Wnt3a and APOC1; (c, d) Migration and invasion in 769P cell intervened by Wnt3a and APOC1. *P < 0.05, **P < 0.01, error bars indicate mean ± SD, t test. [file 12935_2020_1713_MOESM1_ESM.zip › 12935_2021_1713_MOESM3_ESM.tif]

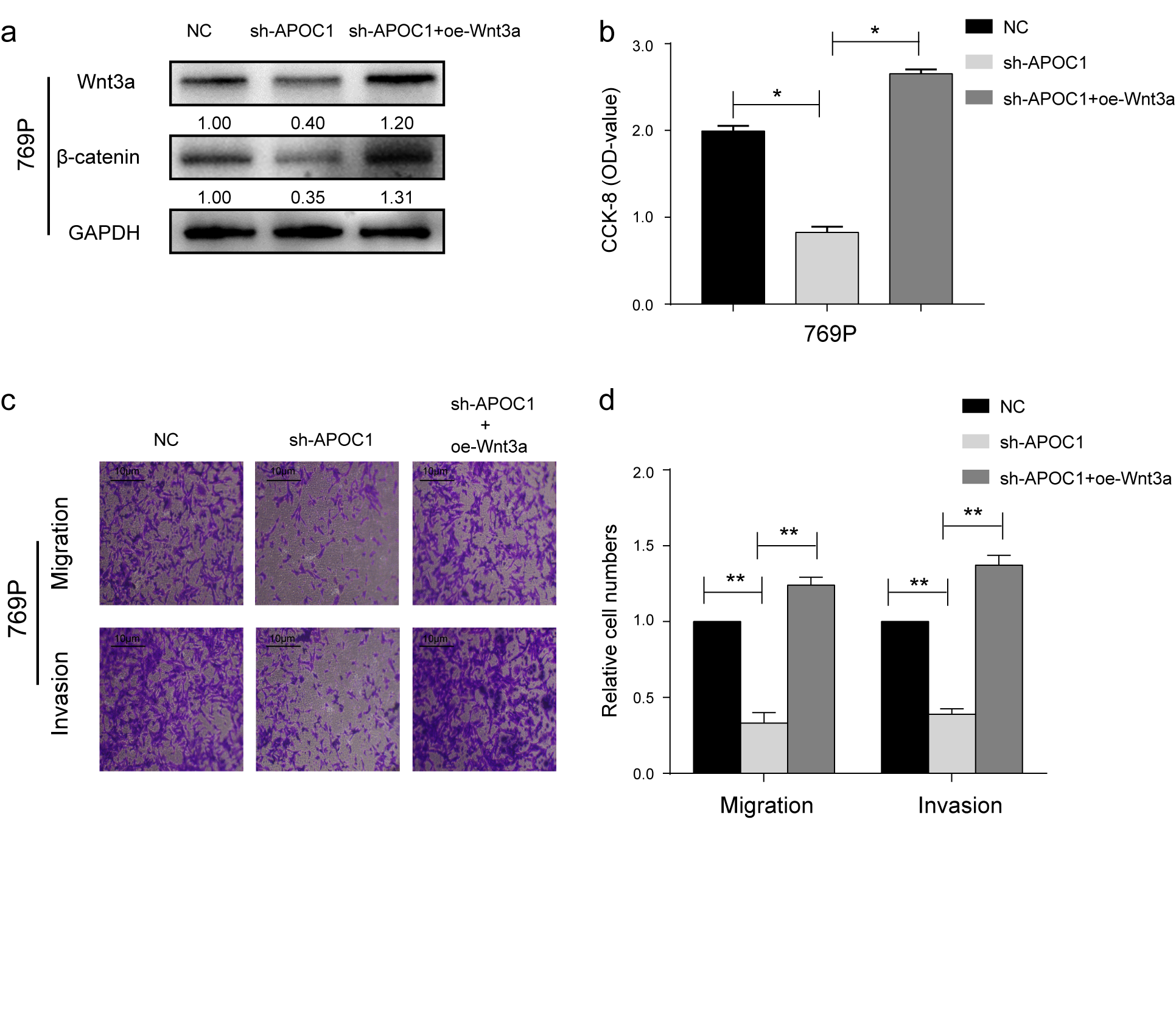

Supplement: Supplementary file 1 — Additional file 1: Figure S1. Relative Protein expression of APOC1 in 12 representative paired cases of RCC and adjacent non-tumoral tissues. (T: RCC tissues, N: adjacent non-tumoral tissues), *P < 0.05, error bars indicate mean ± SD, t test. Figure S2. Relative Protein expression of Wnt3a, β-catenin, CCND1 and TCF7 in CAKI-1 and 769P cell lines with APOC1 knockdown, *P < 0.05, error bars indicate mean ± SD, t test. Figure S3. Relative Protein expression of Wnt3a, β-catenin, CCND1 and TCF7 in CAKI-1 and 769P cell lines overexpressing APOC1, *P < 0.05, error bars indicate mean ± SD, t test. Figure S4. Wnt3a was partially responsible for APOC1-induced aggravation of RCC. (a) Protein level of Wnt3a and β-catenin in 769P cell intervened by Wnt3a and APOC1; (b) Viability in 769P cell intervened by Wnt3a and APOC1; (c, d) Migration and invasion in 769P cell intervened by Wnt3a and APOC1. *P < 0.05, **P < 0.01, error bars indicate mean ± SD, t test. [file 12935_2020_1713_MOESM1_ESM.zip › 12935_2021_1713_MOESM4_ESM.tif]
